# Supplementary material for: PRKG2 Splice Site Variant in Dogo Argentino Dogs with Disproportionate Dwarfism
Source: Genes (Basel). 2021 Sep 24;12(10):1489. doi: 10.3390/genes12101489 (PMC8535654; doi:10.3390/genes12101489)
Supplement: Supplementary file 1 [file genes-12-01489-s001.zip › FigureS1_R1.pdf]

A B

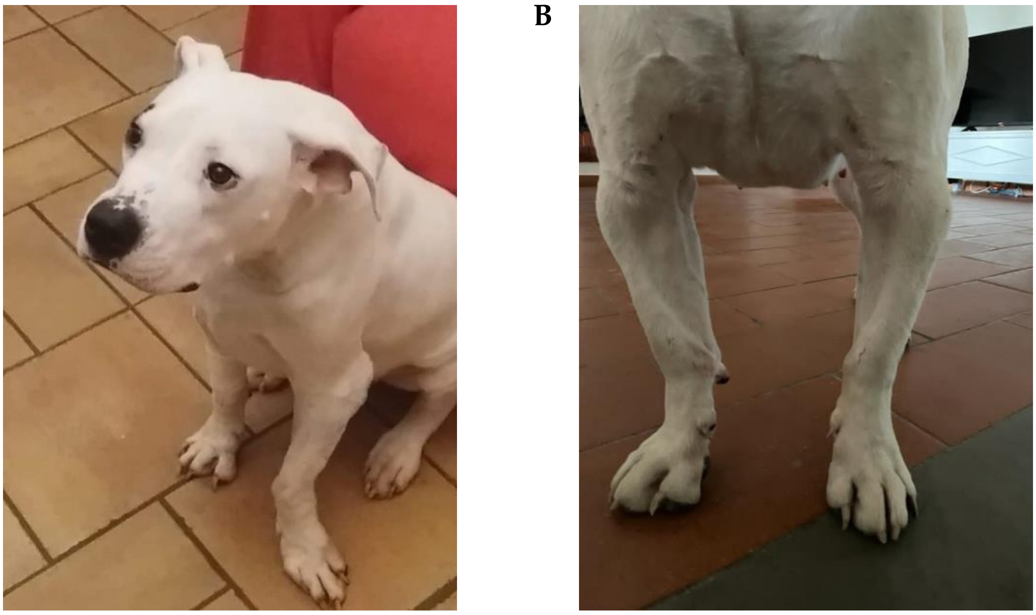

Figure S1. Clinical phenotype of the affected female dog (DG0005). (**A**) This dog had short limbs with angular deformities and an outward rotation of the paws. (**B**) Frontal view of the shortened forelimbs.
